# Insight Into Uncommon Territory: Exploring Internuclear Ophthalmoplegia in Artery of Percheron Infarct

**DOI:** 10.7759/cureus.67485

**Published:** 2024-08-22

**Authors:** Chandni Jayakumar, Nirmala Devi Chandrasekaran, Sritheja Gopalakrishnan, J S Kumar

**Affiliations:** 1 General Medicine, SRM Medical College Hospital and Research Centre, Kattankulathur, IND; 2 General Medicine, SRM Medical College Hospital and Research, Kattankulathur, IND

**Keywords:** medial longitudinal fasciculus, artery of percheron, stroke syndromes, internuclear ophthalmoplegia, thalamic infarction

## Abstract

Internuclear ophthalmoplegia (INO), a neurological disorder is characterized by horizontal gaze palsy because of a lesion in the medial longitudinal fasciculus, a neural pathway that is mainly responsible for coordinating the movements of the eye. INO presents with diplopia and impaired adduction of the affected eye, accompanied by abducting eye nystagmus. The condition also arises from different etiologies which include multiple sclerosis, encephalitis, Lyme disease, HIV, and herpes zoster. Artery of Percheron (AOP) infarction is a subtype of bilateral thalamic infarction that poses a unique form of diagnostic perplexity due to its varied and often non-specific clinical manifestations such as altered responsiveness, memory disturbances, and oculomotor deficits. Here we discuss a 53-year-old female who presented with INO in the context of an AOP infarct. Under this context, the clinical finding includes some paradigms like nystagmus, anisocoria, and bilateral ptosis. Magnetic resonance imaging confirmed an acute infarct in the AOP territory, which supplies the rostral midbrain and paramedian thalami. This case emphasizes the critical importance of meticulous clinical evaluation and the utilization of advanced imaging techniques in diagnosing rare stroke syndromes like AOP infarction. Management of the patient included dual antiplatelet therapy to prevent further thromboembolic events and supportive care to address the immediate neurological deficits. Early recognition and prompt treatment are crucial for better patient outcomes. Long-term management focuses on the secondary prevention of stroke through lifestyle modifications, medical therapy, and regular monitoring. Education on uncommon stroke syndromes and continued research are essential for enhancing the accuracy of diagnosis and efficacy of treatment which ultimately leads to better patient care and prognosis.

## Introduction

Internuclear ophthalmoplegia (INO) results from a lesion in the medial longitudinal fasciculus extending from the abducens nucleus in the pons to the oculomotor nucleus in the midbrain. Drawing in light from the study of Rahmadiansyah et al., this condition often presents with diplopia and impaired horizontal eye movement [1]. Embracing the findings of Hossain et al., even though INO is most commonly associated with multiple sclerosis in younger patients and stroke in older patients, it also results from different neurological conditions, which include neoplasms, infections, and trauma [2].

The artery of Percheron (AOP) is a subtype of bilateral thalamic infarction that presents unique diagnostic challenges because of its varied clinical manifestations. The AOP is a rare structural anomaly where there is a lone arterial trunk that irrigates the paramedian thalami and the rostral midbrain on both sides. Occlusion of this artery can lead to a range of neurological deficits, including altered mental status, vertical gaze palsy, and memory disturbances [3-5].

Given the complex and rare presentation of AOP infarcts, they are often overlooked or misdiagnosed in the process of acute setting [3-6].

This case study analyses a patient presenting with INO in the background of an AOP infarct, highlighting the complexities of neurological localization and the critical importance of thorough clinical evaluation in managing uncommon stroke syndromes.

## Case presentation

A 53-year-old lady with a history of systemic hypertension was brought to the emergency department with decreased responsiveness, eyelid drooping, and difficulty walking for four days. Her vitals upon admission were: BP 160/100 mmHg, pulse rate 92 beats per minute regular rhythm, SPO2 100%, and a Glasgow Coma Scale score of E4V5M6. She was drowsy but could be aroused, was oriented to person and place but not time, and obeyed simple oral commands

**Figure 1:**
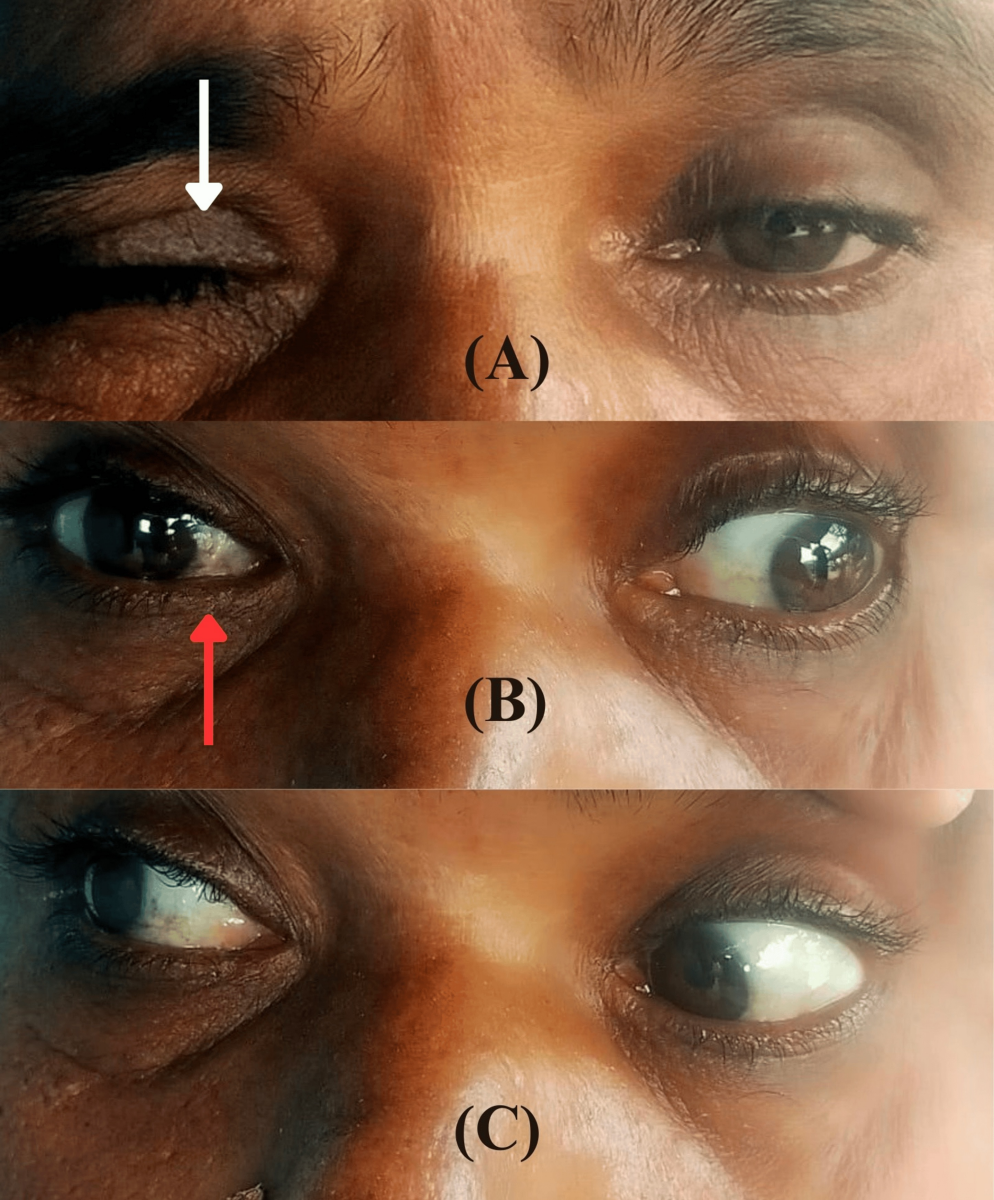
Details of eye examination (A) The image displays unequal drooping of both eyelids and more pronounced ptosis observed on the right side(white arrow). (B) Restricted inward movement of the right eye (red arrow) during leftward gaze. (C) Unaffected ocular movements during rightward gaze.

Upon examination, notable findings included bilateral ptosis, anisocoria, and nystagmus. There was weak adduction of the right eye and abduction nystagmus of the contralateral eye, suggestive of right-side INO. Motor examination revealed bilateral hemiparesis with a power of 4/5 in both upper and lower limbs, normal deep tendon reflexes (DTR), and ongoing plantar reflexes indicative of an upper motor neuron lesion. The eye examination details are depicted in Figure 1.

The biochemical parameter results from blood and CSF are shown in Table 1. 

**Table 1:** Findings from the biochemical analyses of blood and cerebrospinal fluid. Dyslipidemia and impaired fasting glucose observed may contribute to the pathogenesis of the artery of Percheron infarct. CSF: cerebrospinal fluid; HDL: high density lipoprotein; LDL: low density lipoprotein; VLDL: very low density lipoprotein; LDH: lactate dehydrogenase.

PARAMETERS	RESULTS	REFERENCE
BLOOD		
Fasting Glucose	104 mg/dl	70 – 100 mg/dl
LIPID PROFILE	mg/dl	mg/dl
Cholesterol	295	<200
Triglycerides	237	40 – 150
HDL cholesterol	66	> 50
LDL cholesterol	207	80 – 130
VLDL cholesterol	47	<40
RENAL FUNCTION TEST	mg/dl	mg/dl
Urea	43	17 – 43
Creatinine	1	0.6 – 1.2
ELECTROLYTES	mmol/L	
Sodium	134	135 – 145
Potassium	3.8	3.5 – 5.5
Chloride	95	98 – 107
Bicarbonate	27	21 - 31
CSF ANALYSIS		
Opening pressure	10 cm H2O	8 – 18
Appearance	Clear	Clear
Cells	1 – 2 cells (Lymphocytes), No RBCs/WBCs	<5 cells
Proteins	10 mg/dl	<45
Glucose	55 mg/dl	50 – 75
LDH	35 U/L	<40
Chloride	120 mmol/L	118 – 132

**Figure 2:**
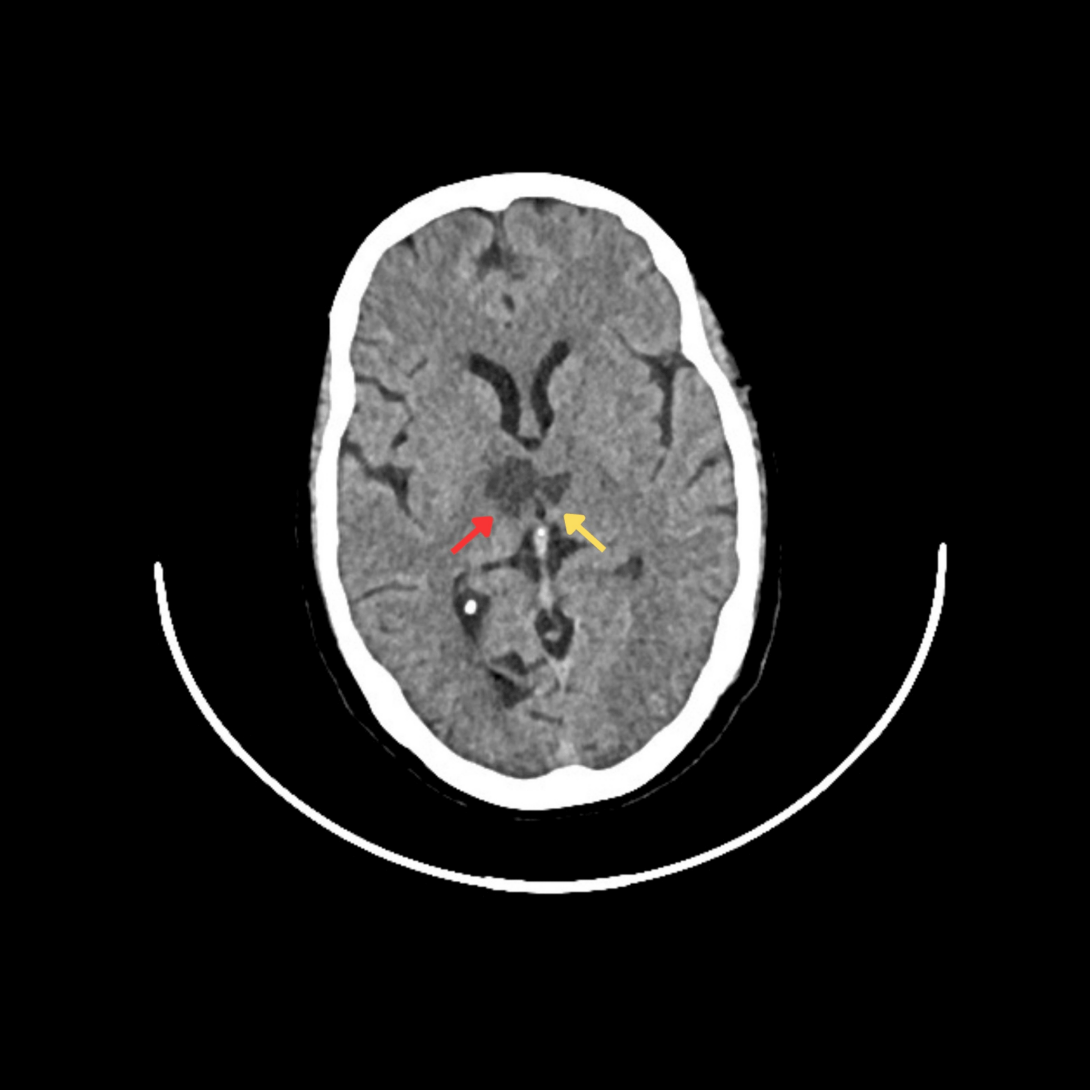
Computed tomography (CT) scan of the brain CT brain axial view displaying distinct area of hypoattenuation in the bilateral paramedial thalamus(right thalamus, red arrow; left thalamus, yellow arrow) advancing into bilateral midbrain resulting in impingement of third ventricle in its posterior aspect.

A computed tomography (CT) scan of the brain was performed, revealing hypodensity in the bilateral paramedian thalamus extending into the midbrain, causing compression of the third ventricle (Figure 2). This imaging finding raised the suspicion of an ischemic event affecting the artery of Percheron.

ECG showed normal sinus rhythm. Echocardiography confirmed no regional wall motion abnormalities, normal left ventricular systolic function, and no LV clot.

**Figure 3:**
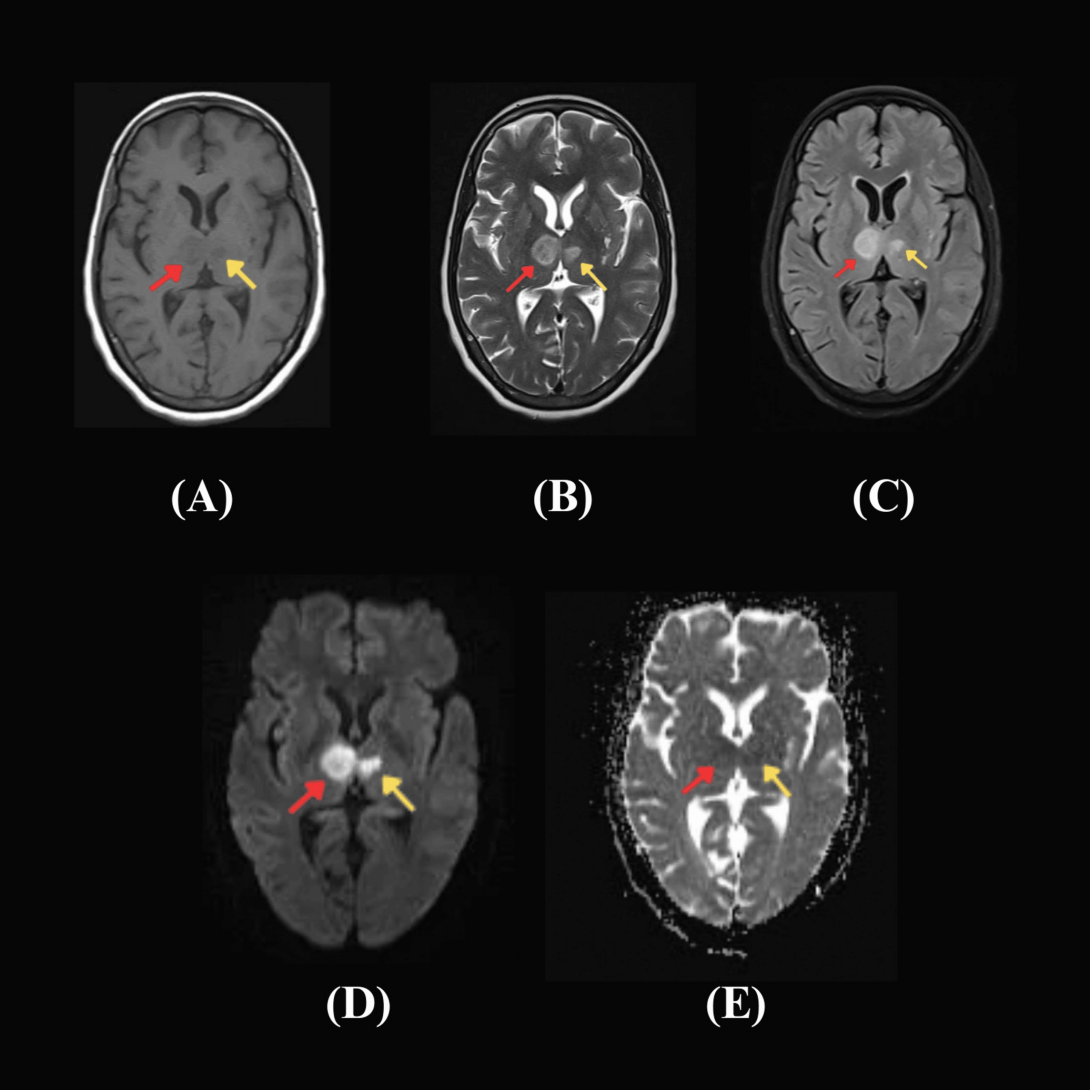
Magnetic resonance imaging (MRI) of the brain MRI findings: (A) - Axial T1: Hypointense signals in both thalami, more on the right. (B)/(C) - Transverse T2/FLAIR: Hyperintense signals in bilateral thalamus, predominantly right, extending to midbrain. (D) and (E) - Diffusion-weighted imaging and Asymmetric diffusion coefficient map: Asymmetric diffusion restriction in bilateral thalamus, primarily on the right side expanding to bilateral midbrain paramedian to midline. Right thalamus, red arrow; left thalamus, yellow arrow. Focal mass effect in the form of mild effacement of the third ventricle – all these features suggestive of acute infarct. FLAIR, fluid-attenuated inversion recovery.

Subsequent magnetic resonance imaging (MRI) of the brain confirmed the presence of an acute infarct. The MRI showed asymmetric diffusion restriction and hyperintense signals in the bilateral medial thalamus extending to the midbrain (Figure 3). These findings were consistent with an infarction in the territory supplied by the artery of Percheron. 

The diverse potential causes of INO were thoroughly investigated, yielding normal results: serum thiamine levels and clinical assessment excluded Wernicke encephalopathy; aquaporin-4 antibodies and cerebrospinal fluid analysis (including viral markers) were negative for Neuromyelitis Optica and encephalitis; imaging studies ruled out arteriovenous malformations and progressive supranuclear palsy; serum vitamin B12 levels and intrinsic factor antibodies were within normal limits excluding pernicious anemia; metabolic testing showed no abnormalities for Fabry disease, maple syrup urine disease, and abetalipoproteinemia; and Lyme disease screening was non-reactive. The acute onset of symptoms, imaging findings, and clinical presentation strongly suggested an ischemic stroke due to an AOP infarct.

The patient was promptly started on dual antiplatelet therapy with aspirin and clopidogrel to prevent further thrombotic events. Close monitoring in the intensive care unit was essential to manage the patient's neurological status and hemodynamic instability following which the patient was subsequently transferred to the ward for ongoing care. She was continued on dual anti-platelets and statins to prevent further thrombotic events. Supportive care including physiotherapy and occupational therapy was initiated to address the motor deficits and promote recovery. Physical therapy sessions targeted her bilateral hemiparesis, emphasizing range of motion exercises such as gait training, and muscle strengthening. Occupational therapy interventions focused on activities of daily living and fine motor skills, while speech therapy addressed swallowing difficulties and communication challenges associated with her initial presentation. Throughout her recovery, close monitoring for complications such as post-stroke seizures and cognitive deficits was monitored with appropriate pharmacological management as needed. Over the subsequent weeks, she demonstrated gradual improvement in consciousness and responsiveness with partial resolution of bilateral ptosis and improvement in ocular motility. There were significant gains in motor function as well. The discharge plan included outpatient rehabilitation services to continue her progress and optimize long-term outcomes.

Given the patient's presentation beyond the therapeutic window for intravenous thrombolysis (tPA) or mechanical thrombectomy, long-term management focused on secondary prevention of stroke. This included the continuation of antiplatelet therapy, optimization of antihypertensive medications to achieve target BP control, and lifestyle modifications to reduce stroke risk factors.

## Discussion

The artery of Percheron, a single dominant arterial trunk arising from one of the proximal segments of the PCA, supplies both paramedian thalami and the rostral midbrain. As mentioned in Kheiralla's study, blockage of this artery is the only variant that results in bilateral paramedian thalamic infarctions with or without midbrain involvement [6].

Infarction in this artery results in a distinct clinical syndrome characterized by a combination of thalamic and midbrain dysfunctions. The thalamus plays a crucial role in sensory relay, motor integration, and consciousness. Infarction in this region may result in a decreased level of alertness ranging from somnolence to akinetic mutism to coma and neuropsychological deficits. In line with the evaluation by Chen et al., Schmahmann JD also noted that AOP infarcts affecting the midbrain can cause oculomotor nerve palsy, resulting in ptosis, anisocoria, and ophthalmoplegia, as observed in this patient. [7,8].

Internuclear ophthalmoplegia occurs due to signal disruption carried by medial longitudinal fasciculus (MLF) signals which are coming from the internuclear abducens and destined for the medial rectus sub-nucleus of the oculomotor nucleus. It is described as a weakness of ipsilateral adduction, especially fractionated or slow adducting saccades (“adduction lag”), and monocular nystagmus in the contralateral abducting eye [9,10].

In this case study, the INO likely resulted from the extension of the infarction from the thalamus into the midbrain affecting the MLF. This features the intricacy of neurological localization and the need for detailed clinical and radiological assessment in stroke patients. Considering the recommendations of Niazi et al., diagnosis of AOP infarct can be arduous due to its varied and non-specific presentations [11]. Just as Carrera et al. cited, bilateral thalamic involvement is uncommon in stroke syndromes and can mimic other conditions [12]. High-resolution imaging modalities such as MRI with diffusion-weighted imaging are crucial for accurate diagnosis. Management of AOP infarct is time-dependent. Timely recognition and treatment with tissue plasminogen activator or mechanical thrombectomy can significantly improve outcomes. In cases presenting beyond the therapeutic window, as in this patient, long-term antiplatelet therapy with statins and risk factor modification is essential to prevent recurrent strokes [13-15]. 

## Conclusions

Ischemic strokes resulting from the artery of Percheron obstruction are an uncommon nature of stroke with unique clinical and radiological features. This case also illustrates the importance and requirement of diligent clinical evaluation and the application of advanced imaging technology in the process of diagnosing and treating several uncommon cerebrovascular accidents. Prompt diagnosis and appropriate treatment are critical in improving patient outcomes. Early administration of thrombolytic therapy or mechanical thrombectomy can be beneficial in eligible patients. For those presenting beyond the therapeutic window, long-term antiplatelet therapy and strict control of vascular risk factors are essential to prevent recurrence.

The wide range of atypical symptoms associated with AOP infarcts should be considered during the evaluation of patients with suspected stroke and clinicians should maintain a high index of suspicion for this rare entity. This case highlights the need for continued research and education on uncommon stroke syndromes to enhance diagnostic accuracy and treatment efficacy.
